# Impact of Continuous Renal Replacement Therapy on Outcomes in Septic Shock Patients Receiving Polymyxin B Hemoperfusion: A Retrospective Cohort Study

**DOI:** 10.3390/biomedicines13122904

**Published:** 2025-11-27

**Authors:** Wei-Hung Chang, Sheng Hsiung Yang, Hsiu-Fang Shen, Ting-Yu Hu, Wen-Jui Wu

**Affiliations:** 1 Department of Critical Care Medicine, MacKay Memorial Hospital, Taipei 10449, Taiwan; peacejaycool@gmail.com; 2 Department of Pulmonary Medicine, MacKay Memorial Hospital, Taipei 10449, Taiwan; 3 Information Department, MacKay Memorial Hospital, Taipei 10449, Taiwan

**Keywords:** sepsis, septic shock, polymyxin B hemoperfusion, continuous renal replacement therapy, vasoactive-inotropic score, endotoxemia, critical care, prognosis

## Abstract

**Background:** Polymyxin B hemoperfusion (PMX-HP) is increasingly used as an adjunctive therapy for severe sepsis and septic shock, yet the prognostic significance of continuous renal replacement therapy (CRRT) and vasoactive-inotropic score (VIS) dynamics under real-world ICU practice remains unclear. This study aimed to evaluate whether CRRT requirement and hemodynamic responses to PMX-HP influence short-term mortality among critically ill patients. **Methods:** We conducted a retrospective cohort study of 64 ICU patients in Taiwan with severe sepsis or septic shock who received PMX-HP. Clinical characteristics, illness severity, VIS measurements before and after PMX-HP, organ-support therapies, and outcomes—including 28-day mortality, ICU and hospital mortality, and lengths of stay—were analyzed. Patients were stratified by CRRT use, and multivariate logistic regression was performed to identify independent predictors of 28-day mortality. **Results:** Among 64 patients (mean age 66 years; 67% male), 67.2% received CRRT and the overall 28-day mortality was 46.9%. CRRT users exhibited higher crude mortality and higher APACHE II scores. Survivors were younger and had lower baseline severity. Hemodynamic trajectories differed substantially: VIS increased after PMX-HP more frequently in non-survivors than survivors. In multivariate analysis, post-PMX-HP VIS elevation and higher APACHE II were independent predictors of 28-day mortality, whereas CRRT requirement was not an independent determinant. **Conclusions:** In this real-world cohort, PMX-HP did not significantly reduce mortality. Illness severity and inadequate vasopressor improvement, rather than CRRT use, primarily determined outcomes. VIS elevation following PMX-HP may serve as an early indicator of poor hemodynamic recovery in septic shock.

## 1. Introduction

Severe sepsis and septic shock remain major challenges in intensive care units (ICUs) worldwide, with high mortality despite improvements in early recognition and standardized management [1]. The Surviving Sepsis Campaign guidelines (2016 and 2021) emphasize prompt antimicrobial therapy, source control, and hemodynamic support [1,2], yet outcomes for patients in refractory shock with multi-organ failure remain poor. The 2016 Sepsis-3 consensus redefined septic shock (vasopressor-dependent hypotension with lactate > 2 mmol/L) to identify patients at particularly high risk of death [3]. Globally, sepsis caused an estimated 48.9 million cases and 11 million deaths in 2017, with especially high mortality in resource-limited settings [4]. In Taiwan, nationwide data show rising sepsis incidence and persistently high acute mortality over the past two decades [5,6]. These statistics underscore the urgent need for more effective therapies to improve septic shock outcomes. Recent multinational ICU data, global health priority statements, and time-to-treatment outcome analyses further highlight the worldwide burden and urgency of sepsis management [7,8,9].

Endotoxin (lipopolysaccharide, LPS) is a central driver of the dysregulated immune response in Gram-negative sepsis. LPS triggers a cascade of inflammatory signaling via Toll-like receptor 4, leading to a “cytokine storm”, endothelial injury, capillary leak, and vasodilatory shock that collectively impair tissue perfusion and precipitate multi-organ failure. Notably, endotoxemia can also occur in some patients with non–Gram-negative infections (e.g., fungal or viral sepsis), possibly due to loss of gut barrier integrity and bacterial translocation during critical illness. These insights have prompted the development of extracorporeal blood purification therapies to remove endotoxin and other inflammatory mediators from the circulation as an adjunct to conventional sepsis management.

Polymyxin B hemoperfusion (PMX-HP) is one such therapy, designed to directly remove circulating endotoxin using an immobilized polymyxin B column. By neutralizing endotoxin extracorporeally, PMX-HP avoids the nephrotoxicity and neurotoxicity of systemic polymyxin. Since its introduction, PMX-HP has been widely used in parts of Asia (notably Japan and Taiwan) for refractory Gram-negative septic shock. Early case series reported improved blood pressure and reduced vasopressor requirements following PMX-HP, fueling enthusiasm for the technique [10]. However, the efficacy of PMX-HP in broader septic shock populations remains controversial. Large randomized trials to date have not demonstrated a significant survival benefit. In the EUPHRATES trial, which targeted patients with high endotoxin activity, PMX-HP failed to significantly reduce 28-day mortality compared to sham treatment (39% vs. 43%) [11]. Similarly, a 2024 meta-analysis concluded that while PMX-HP often improves hemodynamics, it does not significantly lower overall mortality [12]. These mixed findings temper the initial enthusiasm [10] and suggest that any benefit of PMX-HP may be limited to specific high-risk scenarios rather than indiscriminate use in all septic shock patients.

In addition to pathogen-targeted therapies, the trajectory of a patient’s hemodynamics provides important prognostic information. The vasoactive–inotropic score (VIS) quantifies the total vasopressor/inotrope dose required to support circulation. A high VIS reflects severe vasodilatory shock requiring multiple vasopressors, and a rising VIS indicates refractory shock. Recent research has identified VIS as an early predictor of mortality in sepsis; for example, adult septic patients with a VIS > 30 in the first 6 h of ICU care had over 50% mortality. We postulated that failure of vasopressor requirements to decrease (i.e., an increasing VIS) after PMX-HP therapy would signal a poor prognosis.

Another critical aspect of septic shock management is organ support for failing organs. Acute kidney injury (AKI) is common in septic shock and often necessitates renal replacement therapy [1,13]. Continuous renal replacement therapy (CRRT) is a mainstay for hemodynamically unstable patients with severe AKI, allowing gentle fluid removal and clearance of metabolic wastes. There is interest in whether CRRT—especially using high-cutoff or adsorptive membranes—can modulate the septic inflammatory response by filtering cytokines and endotoxin. A recent systematic review reported that septic patients treated with CRRT using an adsorptive oXiris filter had lower 28-day mortality and reduced vasopressor requirements compared to standard CRRT (though the evidence quality was low) [14]. Conversely, the optimal timing of CRRT in septic AKI remains debated. A 2025 meta-analysis of trials found that initiating CRRT very early (at the first sign of AKI) did not improve 28- or 60-day mortality compared to a delayed strategy, and early initiation was associated with higher rates of complications such as hypotension [15]. These findings suggest that while CRRT is indispensable for managing established organ failure, it is not a cure for sepsis itself—its benefit likely lies in preventing fluid overload and electrolyte crises rather than directly improving survival.

The main aim of this study was to evaluate the impact of continuous renal replacement therapy (CRRT) on clinical outcomes—including 28-day mortality, hemodynamic changes assessed by vasoactive-inotropic score (VIS), and organ support requirements—in critically ill patients with septic shock receiving polymyxin B hemoperfusion (PMX-HP).

## 2. Materials and Methods

Study Design and Setting: This study is a single-center retrospective cohort analysis conducted in the medical ICU of MacKay Memorial Hospital, a 25-bed tertiary care ICU in Taipei, Taiwan. The study period spanned from 1 July 2013 to 31 December 2019. The ICU provides comprehensive care for critically ill adults, including advanced life support and extracorporeal therapies. The study was approved by the hospital’s Institutional Review Board (IRB No. 18MMHIS198e), which waived informed consent due to the observational, de-identified nature of the research.

**Figure 1:**
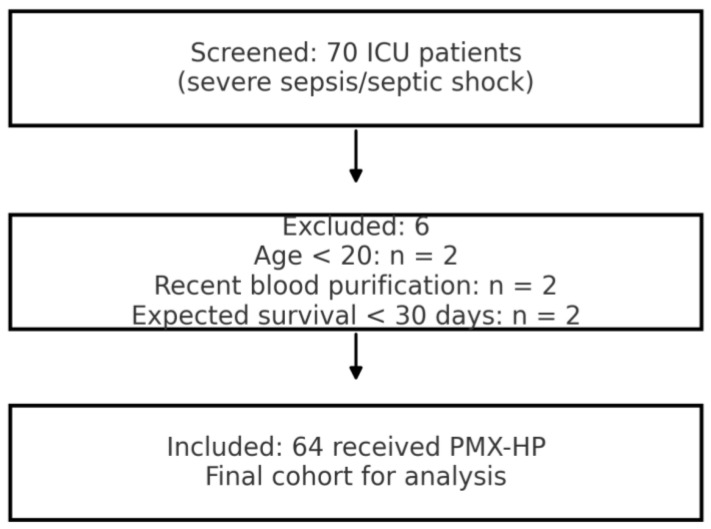
Flow diagram of patient enrollment and exclusion. Abbreviations: ICU, intensive care unit; PMX-HP, polymyxin-B hemoperfusion.

Patient Population: We screened all patients admitted to the ICU during the study period with a diagnosis of sepsis or septic shock. We applied the Sepsis-3 definition for septic shock (2016 criteria)—i.e., sepsis with persistent hypotension requiring vasopressors to maintain mean arterial pressure ≥65 mmHg and having a serum lactate >2 mmol/L despite adequate fluid resuscitation [3]. Inclusion criteria were: (1) age ≥ 20 years; (2) diagnosis of severe sepsis or septic shock (per above) due to any infectious etiology; and (3) treatment with at least one session of PMX-HP during the ICU stay. Patients were excluded if they met any of the following: pregnancy; participation in another interventional trial within 1 month; recent organ transplantation (within 1 year); advanced malignancy or other condition with anticipated survival <30 days (as determined by the treating physician); cardiopulmonary resuscitation within 4 weeks prior to ICU admission; do-not-resuscitate (DNR) order or comfort care status at admission; known HIV infection; uncontrolled active bleeding at ICU admission; brain death; end-stage liver disease (Child-Pugh class C); recent use of other extracorporeal blood purification therapies (e.g., continuous venovenous hemofiltration, hemodialysis, plasma exchange) within 24 h before PMX-HP; known hemophilia or bleeding diathesis; or a history of allergy to polymyxin B, heparin, or extracorporeal circuit materials. Among the inclusion and exclusion criteria, a total of 70 patients were initially identified. Among them, 6 were excluded—2 minors, 2 with recent prior blood purification therapy, and 2 with an expected <30-day survival due to advanced cancer—resulting in a final study cohort of 64 patients (Figure 1).

Data Collection: We retrospectively collected clinical data from electronic medical records using a standardized case report form. Data included: demographics (age, sex, body weight); comorbidities (e.g., diabetes, hypertension, chronic kidney disease); source of infection (e.g., pneumonia, intra-abdominal, urinary tract, etc.); causative pathogens isolated; illness severity scores (Acute Physiology and Chronic Health Evaluation II, APACHE II) at ICU admission; vasopressor and inotrope usage; organ support measures; and outcomes. APACHE II scores were calculated based on the worst physiological values in the first 24 h of ICU admission. We recorded the details of PMX-HP therapy for each patient: number of sessions (usually 1 or 2, but up to 4 if repeated), duration of each hemoperfusion session, and whether sessions were given sequentially (two sessions within 24 h) or on separate days. For CRRT, we noted whether and when it was initiated relative to shock onset (categorized as within 24 h of shock vs. later in ICU stay). Use of extracorporeal membrane oxygenation (ECMO) for refractory respiratory or cardiac failure was also recorded.

We paid particular attention to vasoactive-inotropic score (VIS) as a marker of hemodynamic support [16]. The VIS was calculated at three time points corresponding to the typical PMX-HP treatment timeline in our ICU: just before the first PMX-HP session (T1, baseline), immediately after the first PMX-HP session (T2), and 24 h after the first PMX-HP (T3). The VIS was defined as:

*VIS* = *dopamine* (μg/kg/min) + *dobutamine* (μg/kg/min) + 100 × *epinephrine* (μg/kg/min) + 100 × *norepinephrine* (μg/kg/min) + 10 × *milrinone* (μg/kg/min) + 10,000 × *vasopressin* (U/kg/min).

This formula assigns weighted points to each vasopressor/inotrope based on potency. A higher VIS indicates a higher dose and/or number of vasoactive drugs. We then derived the change in VIS across PMX-HP: ΔVIS = VIS at 24 h post-PMX (T3) minus VIS immediately post-PMX (T2). A positive ΔVIS thus signifies that vasopressor requirements increased in the 24 h after PMX-HP, whereas a negative ΔVIS indicates a decrease (improvement in hemodynamic status). We categorized patients as having an “improved” hemodynamic status if VIS decreased or went to zero after therapy, versus “no improvement or worsening” if VIS stayed the same or rose (ΔVIS ≥ 0).

PMX-HP Therapy: PMX-HP was performed using the Toraymyxin^®^ PMX cartridge (Toray Industries, Tokyo, Japan), following our ICU’s standard protocol. Vascular access was via a dialysis catheter (typically femoral or internal jugular). Anticoagulation of the circuit was achieved with systemic heparin or citrate per our standard dialysis protocol unless contraindicated by bleeding risk. Each PMX-HP session was prescribed for ~2 h (some extended to 6 h at physician discretion). Blood flow rates were generally 80–120 mL/min. In most cases, patients received two PMX-HP sessions 24 h apart, in line with common practice and National Health Insurance reimbursement (which covers up to two sessions). The decision to administer a single session or more than two sessions was at the treating intensivist’s discretion based on patient response. Sequential therapy (two cartridges back-to-back in one day) was occasionally used for fulminant cases. All patients continued to receive standard sepsis care including antibiotics, fluid resuscitation, vasopressors, mechanical ventilation, and source control as needed, in accordance with SSC guidelines.

CRRT and Other Organ Support: CRRT was available continuously in our ICU and was initiated for patients who developed severe AKI (typically oliguria/anuria with volume overload, or AKI with severe acidemia or hyperkalemia) or in cases of refractory fluid overload not responsive to diuretics. The modality was continuous venovenous hemofiltration or hemodiafiltration, using high-flux membranes (note: during the study period, oXiris filters were not yet routinely used in our unit). CRRT settings (blood flow ~150–200 mL/min, dose ~20–25 mL/kg/hour effluent) were per standard practice. In some instances, CRRT was also used to facilitate fluid removal in patients on ECMO or with congestive cardiac failure. We documented the timing of CRRT initiation relative to vasopressor start: early (within 24 h of shock onset) vs. delayed. ECMO (veno-arterial or veno-venous) was instituted in a small number of patients with either refractory cardiogenic shock or severe respiratory failure (PaO_2_/FiO_2_ < 80 on maximal ventilator support), following multidisciplinary approval.

Outcome Measures: The primary outcome for this study was 28-day all-cause mortality, defined as death from any cause within 28 days after the first PMX-HP session. We also recorded ICU mortality (death before ICU discharge) and hospital mortality (death before ultimate hospital discharge). Secondary outcomes included ICU length of stay and hospital length of stay (days), and organ support duration (days of mechanical ventilation, CRRT, etc.), where available. We specifically examined the changes in vasoactive support (VIS) around PMX-HP as described, to see if PMX-HP was associated with a reduction in vasopressor requirements, and whether that correlated with survival. Finally, we assessed factors associated with mortality, including patient demographics, severity scores, infection characteristics, and use of therapies like CRRT.

Statistical Analysis: Data were analyzed using IBM SPSS Statistics version 26 (IBM Corp., Armonk, NY, USA). Continuous variables are presented as mean ± standard deviation (SD) if approximately normally distributed, or median with interquartile range (IQR) if non-normal. Categorical variables are summarized as counts and percentages. We used the Kolmogorov–Smirnov test to assess normality of distributions. For comparisons between groups (e.g., survivors vs. non-survivors, or CRRT vs. no-CRRT patients), we employed Student’s *t*-test for normally distributed continuous data and the Mann–Whitney *U* test for skewed data. Chi-square or Fisher’s exact tests (when expected cell counts were <5) were used for categorical variables. A two-tailed *p* < 0.05 was considered statistically significant.

To identify prognostic factors for 28-day mortality, univariate logistic regression was first performed for candidate variables including age, sex, APACHE II score, need for CRRT (within 24 h and within 28 d), need for ECMO, number of PMX sessions, use of sequential PMX, baseline lactate, and VIS change (ΔVIS positive vs. negative). Variables with *p* < 0.10 in univariate analysis or with strong clinical plausibility were entered into a multivariate logistic regression model to determine independent predictors of mortality. Adjusted odds ratios (OR) with 95% confidence intervals (CI) were calculated. Model fit was checked with the Hosmer-Lemeshow goodness-of-fit test. No variable had >5% missing data; thus, a complete-case analysis was used. We did not impute missing values given the small sample size. In subgroup analysis, we stratified the cohort by CRRT usage at any time and compared outcomes between those who received CRRT vs. those who did not, using the same statistical tests as above.

## 3. Results

**Figure 2:**
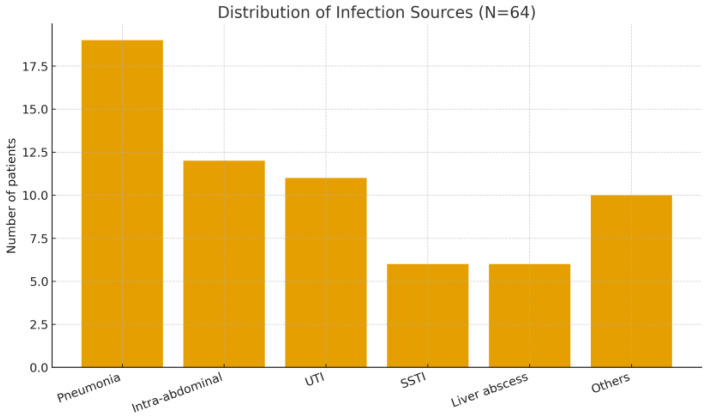
Distribution of infection sources among patients receiving PMX-HP (N = 64). Abbreviations: UTI, urinary tract infection; SSTI, skin/soft tissue infection.

**Figure 3:**
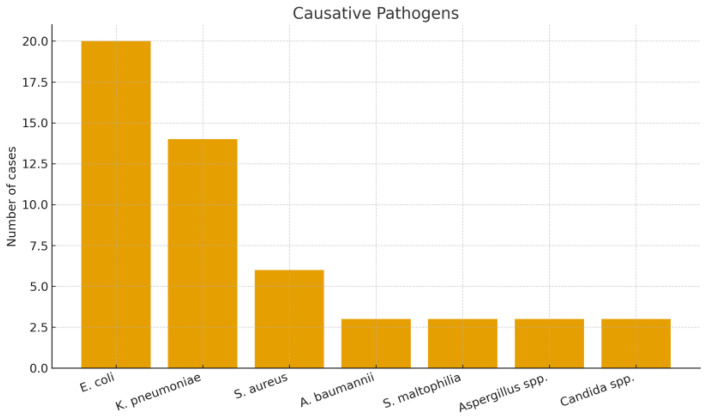
Distribution of causative pathogens among patients receiving PMX-HP.

Patient Flow and Cohort Description: During the 6.5-year study period, a total of 70 ICU patients met the initial screening criteria for sepsis or septic shock requiring PMX-HP. After applying the inclusion and exclusion criteria, 6 patients were excluded (2 minors, 2 with recent prior blood purification therapy, and 2 with expected <30-day survival due to advanced cancer). The remaining 64 patients constituted our final study cohort (Figure 1). Baseline characteristics of the 64 patients are summarized in Table 1, demonstrating a predominantly elderly, critically ill population with high illness severity and diverse infection sources. The mean age was 66.1 years (SD ± 12.3, range 37–94) and 67.2% were male. The cohort was critically ill on presentation, with a median APACHE II score of 26 (IQR 21–32); notably, 95% of patients had an APACHE II > 12, reflecting at least moderate to severe illness. The most common sources of infection were pneumonia (19 patients, 29.7%),followed by intra-abdominal infections such as peritonitis or abscess (12 patients, 18.8%), urinary tract infections/urosepsis (11 patients, 17.2%), and skin/soft tissue infections including necrotizing fasciitis (6 patients, 9.4%). We also had 6 patients (9.4%) with liver abscesses (a notable entity in East Asia), and a few cases of primary bacteremia, a brain abscess, post-trauma sepsis, and one case of fungemia. Figure 2 shows the distribution of infection sources graphically. Microbiologically, Gram-negative bacteria were predominant: Escherichia coli was isolated in 31.3% of patients and Klebsiella pneumoniae in 21.9%, making these the leading pathogens (often in intra-abdominal and urinary sources). Staphylococcus aureus (9.4%) was the most common Gram-positive organism (mostly in skin/soft tissue infections), with a minority of cases caused by less common Gram-negative or fungal pathogens (Figure 3). Blood cultures were positive in approximately half of patients. Comorbid conditions were frequent: 53% had diabetes mellitus, 59% hypertension, 11% chronic kidney disease (stage 3–5), and 3% were on chronic dialysis for end-stage renal disease.

All 64 patients received at least one PMX-HP session as per inclusion criteria. Most patients (51 out of 64, 79.7%) underwent the standard two PMX-HP sessions 24 h apart. Nine patients (14.1%) received only a single session (due to early death or a clinical decision that the patient improved or was too unstable for a second session). Two patients (3.1%) received a third session, and another two (3.1%) received four sessions (in these cases, additional PMX treatments were given due to persistent septic shock and high clinical suspicion of ongoing endotoxemia). The majority (87.5%) of PMX-HP sessions were ~2 h in duration as per protocol. In about half the patients (51.6%), the two PMX-HP sessions were given in a sequential manner (within a single day or consecutive days); the remaining had non-sequential sessions (e.g., 24–48 h apart). No immediate adverse events from PMX-HP (e.g., allergic reactions, severe hypotension beyond expectations) were noted in the records.

Clinical Outcomes: Key outcomes for the cohort are presented in Table 2. The primary outcome, 28-day mortality, was 46.9% (30 of 64 patients). ICU mortality (death before ICU discharge) was 53.1%, and in-hospital mortality was also 53.1% (34 of 64 patients died before hospital discharge). The concordance of ICU and hospital mortality reflects that most deaths occurred early during critical illness; only 4 patients died after ICU discharge but before hospital discharge. The median ICU length of stay (LOS) for the whole cohort was 9.3 days (IQR 4.4–21.1 days). Surviving patients often had prolonged hospital stays for ongoing recovery and rehabilitation, with a median hospital LOS of 20.5 days (IQR 8.0–34.6 days). Among survivors, many required stepped-down care after ICU (e.g., high-dependency or general ward care for several weeks). The median duration of mechanical ventilation was approximately 10 days for those who were intubated. Nearly all patients (>90%) required invasive mechanical ventilation during their ICU course, given the severity of their illnesses.

CRRT was a commonly utilized therapy in this cohort. Overall, 43 patients (67.2%) received CRRT at some point during their ICU stay. Importantly, 45.3% of patients had CRRT initiated within the first 24 h of shock onset (i.e., early CRRT, often started concurrently with PMX-HP), reflecting AKI or shock-related oliguria early in their course. By 28 days, additional patients had CRRT started later, bringing the total of who ever required CRRT to 67.2%. CRRT was typically continued for days to weeks as needed; among survivors who received CRRT, a subset eventually recovered kidney function, whereas others remained dialysis-dependent at discharge. ECMO was used in 3 patients (4.7%); 2 of these were veno-arterial ECMO for refractory cardiogenic shock (one due to septic cardiomyopathy, one due to a concurrent myocardial infarction), and 1 was veno-venous ECMO for ARDS due to pneumonia. Despite these aggressive measures, all 3 ECMO patients died (2 in ICU, 1 shortly after ICU discharge).

Comparison of Survivors and Non-Survivors: We compared baseline and treatment variables between 28-day survivors (n = 34) and non-survivors (n = 30) to identify factors associated with mortality (Table 3). Non-survivors were older on average than survivors (mean 68.5 ± 9.8 years vs. 63.4 ± 14.3 years), although this difference did not reach conventional significance (*p* = 0.098). There was a significant sex difference: 79.4% of non-survivors were male, compared to 53.3% of survivors (*p* = 0.03), suggesting male sex may have been a risk factor or coincided with more severe illness. Illness severity was notably higher in non-survivors, with mean APACHE II score 27.5 ± 6.5 vs. 24.2 ± 6.0 in survivors (*p* = 0.049). In fact, 70% of non-survivors had APACHE II > 25, whereas survivors more often had scores in the high-teens to low-20s. There was no significant difference in body weight or most comorbidities between the groups, except that chronic kidney disease was slightly more prevalent in non-survivors (13.3% vs. 8.8%), although numbers were small. The distribution of infection sources did not differ appreciably by outcome, and both groups had similar proportions of Gram-negative vs. Gram-positive pathogens. Treatment variables showed clearer differences.

**Figure 4:**
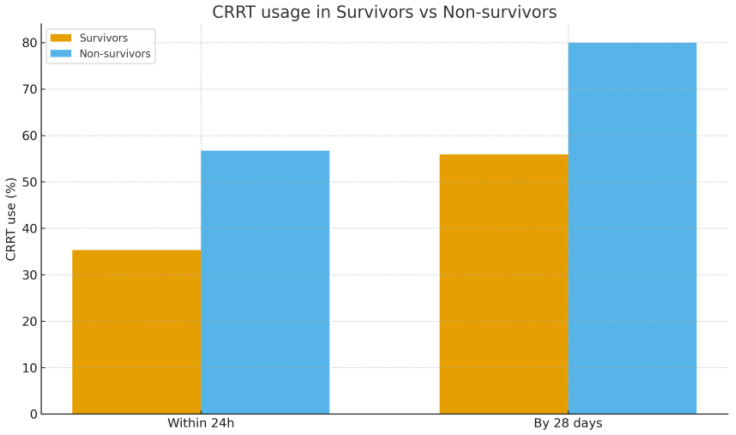
Comparison of CRRT usage between survivors and non-survivors within 24 h and by 28 days after shock onset. Abbreviations: CRRT, continuous renal replacement therapy.

Treatment variables showed clearer differences. All patients by definition received PMX-HP, and the median number of PMX sessions was 2 in both groups. Non-survivors were slightly more likely to have received only 1 session (likely due to early demise), but this was not significant. The use of sequential PMX (two cartridges in 24 h) versus spaced sessions was comparable between survivor and non-survivor. Crucially, non-survivors required significantly more organ support. Within the first 24 h of septic shock onset, 55.9% of non-survivors had CRRT started, compared to 33.3% of survivors (*p* = 0.023). By 28 days, 82.4% of non-survivors had needed CRRT at some point vs. 50.0% of survivors (*p* < 0.01). In other words, patients who died were 2–4 times more likely to require dialysis for AKI than those who survived. This yields an unadjusted odds ratio of approximately 4.7 for mortality associated with the need for CRRT (within 28 days) in our cohort. Figure 4 illustrates the difference in CRRT utilization between survivors and non-survivors. As for ECMO, 2 non-survivors vs. 1 survivor received ECMO (5.9% vs. 3.3%, *p* = 0.63). ICU LOS were similar (median ~9–10 days in both groups, *p* = 0.78); many non-survivors died early, but a few had very prolonged ICU courses before eventual death, balancing the median. Survivors who recovered, on the other hand, often stayed longer in the hospital post-ICU (Accordingly the significant difference in total hospital LOS: median 27.9 days for survivors vs. 13.0 days for non-survivors, *p* = 0.007).

CRRT vs. No-CRRT Subgroup Analysis: Given the strong association between CRRT use and mortality, we further analyzed patients stratified by whether they received CRRT during the ICU stay (regardless of survival). Out of 64 patients, 43 (67.2%) received CRRT and 21 (32.8%) did not. Patients who required CRRT had more severe illness on admission and worse outcomes. The CRRT group had a higher median APACHE II score (26.5, IQR 23–33) compared to the no-CRRT group (24.0, IQR 20–27; *p* = 0.03). They were also more likely to be male (76.7% vs. 47.6%, *p* = 0.02). The 28-day mortality in the CRRT group was 58.1% (25/43) versus 23.8% (5/21) in the no-CRRT group (*p* = 0.008). This finding reflects that patients not requiring CRRT were generally those without severe AKI, who had a better chance of survival. Among patients who never needed CRRT, most had lower vasopressor requirements, and some stabilized with source control and antibiotics alone. In contrast, the CRRT group included the sickest organ failure. We did not find significant differences in the infection source or organism profile between the CRRT and non-CRRT subgroups; the key distinguishing factors were the severity of illness and ensuing organ failures.

**Figure 5:**
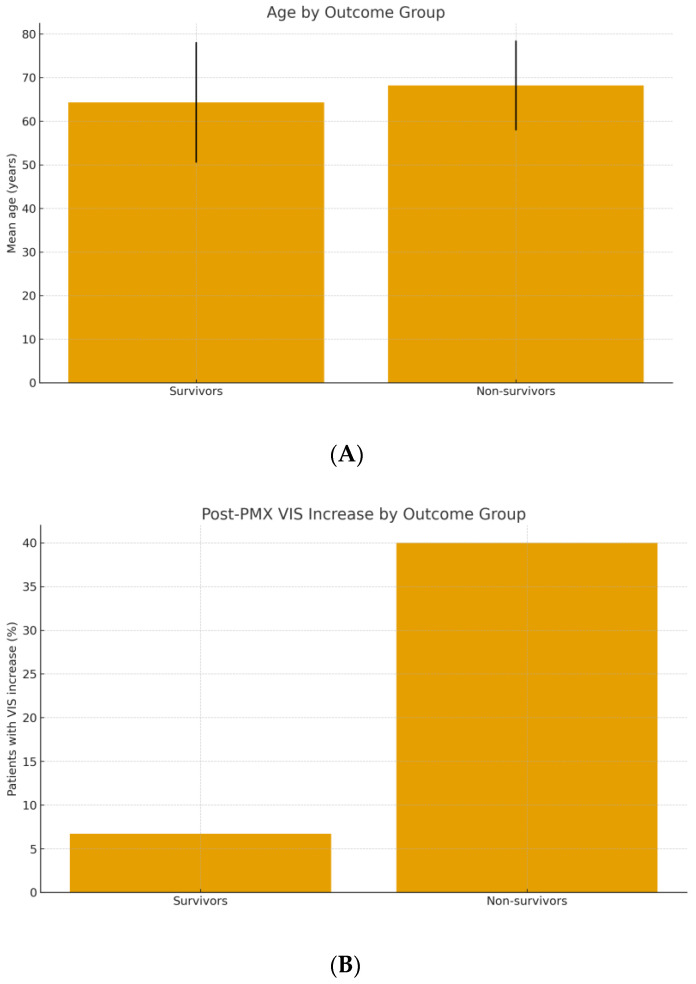
(**A**) Mean age of survivors vs. non-survivors treated with PMX-HP (error bars represent ±1 SD). (**B**) Incidence of a post-treatment increase in vasoactive-inotropic score (VIS) in survivors vs. non-survivors.

Hemodynamic Response and VIS Dynamics: We assessed the acute hemodynamic changes around PMX-HP therapy using the VIS. Overall, 60 of 64 patients were on vasopressors at the time of PMX-HP initiation; the remaining 4 had received vasopressors earlier but were stabilized enough to come off briefly before PMX-HP. The median VIS just before PMX-HP (T1) was 20 (approximately equivalent to norepinephrine ~0.2 μg/kg/min, or an equivalent combination of agents). Immediately after the PMX-HP session (T2), there was no uniform trend in VIS: some patients had decreased vasopressor requirements, others remained the same or even required more support during the treatment due to labile hemodynamics. By 24 h post-PMX (T3), 39 patients (60.9%) showed a decrease in VIS compared to baseline, indicating improved blood pressure and reduced vasopressor dependence. In contrast, 25 patients (39.1%) had no improvement or an increase in VIS after PMX-HP, reflecting ongoing or worsening shock. Crucially, this hemodynamic trajectory was associated with outcome. Among non-survivors, 35.3% experienced a rise in VIS from immediately post-treatment to 24 h later, compared to only 6.7% of survivors (*p* = 0.006) (Table 3). Conversely, the majority of survivors had a significant drop in VIS after PMX-HP (often enabling tapering off of vasopressors within 1–2 days), whereas many who died could not be weaned off supports. This suggests that failure to respond hemodynamically to PMX-HP (or to overall therapy) predicted a poor prognosis. Figure 5 illustrates the difference in mean post-treatment VIS between survivors and non-survivors. The positive ΔVIS in a subset of patients likely indicates ongoing uncontrolled inflammatory vasoplegic shock, for which PMX-HP and standard measures were insufficient.

Prognostic Factors for Mortality: Table 4 presents the results of univariate and multivariate logistic regression for 28-day mortality. On univariate analysis, factors significantly associated with higher odds of death included: higher APACHE II score (per point increase, OR 1.10, 95% CI ~1.01–1.19, *p* = 0.03); CRRT requirement within 24 h (OR 2.53, 95% CI 1.13–5.65, *p* = 0.025); CRRT requirement by 28 days (OR 4.67, 95% CI 1.56–13.99, *p* = 0.006); and a positive ΔVIS (i.e., VIS increase after PMX-HP, OR ~7.8, *p* = 0.004). Male sex had an OR of 3.50 (*p* = 0.03). Older age trended toward significance (OR ~1.04 per year, *p* = 0.10). In multivariate analysis, we constructed a model including age, APACHE II, sex, CRRT (by 28 d), and ΔVIS category. In the final adjusted model, only two factors remained independent predictors of 28-day mortality: an increase in VIS after PMX-HP (adjusted OR ~7.5, *p* = 0.01) and APACHE II score (adjusted OR ~1.12 per point, *p* = 0.04). The model had a good fit (Hosmer–Lemeshow *p* = 0.82) and an area under the ROC curve of 0.81 for mortality prediction. These results highlight that patients who died were those with more severe acute illness (high APACHE II), who developed severe organ failure requiring dialysis, and who did not achieve hemodynamic improvement even after adjunctive therapy.

## 4. Discussion

Our real-world data show that despite the use of PMX-HP in a cohort of very ill septic shock patients, mortality remained around 50%. The need for CRRT was clearly a marker of illness severity and was associated with worse unadjusted outcomes, but it was not an independent predictor of mortality in adjusted analysis. By contrast, failure of vasopressor requirements to decline (reflected by a rising VIS after therapy) was strongly associated with mortality.

In this single-center cohort of refractory septic shock patients treated with PMX-HP, 28-day mortality was approximately 47%. This outcome reflects the high severity of illness in our population and is consistent with the substantial mortality reported in contemporary septic shock studies, even with guideline-directed care [1]. Notably, no adjunctive therapy to date has achieved a dramatic reduction in sepsis mortality or significantly improved long-term outcomes [17]. Multiple interventions tested in the past decade have yielded inconclusive results. For example, prolonged low-dose corticosteroid therapy did not significantly reduce mortality in large septic shock trials [18,19]; resuscitation guided by peripheral perfusion targets (capillary refill time) conferred no survival advantage over lactate-guided resuscitation [20]; and the novel vasopressor angiotensin II improved blood pressure but not 28-day mortality in vasodilatory shock [21]. Even extracorporeal endotoxin removal with PMX-HP failed to show an overall survival benefit except perhaps in highly selected scenarios [22]. Likewise, observational analyses indicate that overall sepsis mortality has remained persistently high in recent years [23]. In parallel, a network meta-analysis of blood purification techniques found no clear improvement in survival with these extracorporeal therapies [24]. Taken together, these findings underscore that, despite incremental advances, septic shock continues to have high acute mortality.

Some progress has been made in optimizing fundamental supportive care for septic shock. Recent trials support using balanced crystalloid solutions instead of saline, avoiding excessive fluid overload, and tolerating a slightly lower blood pressure target in older patients to minimize iatrogenic harm [25,26,27]. For instance, the use of buffered crystalloids in ICU fluid resuscitation has been associated with improved renal outcomes over normal saline, and a conservative fluid strategy in established septic shock was shown to be safe and potentially reduced complications. Additionally, permitting a lower mean arterial pressure goal (e.g., MAP 60–65 mmHg) in vasodilatory hypotension for patients over 65 years (the “65 trial”) did not increase mortality and led to less vasopressor exposure [26]. Despite adherence to such best practices, once severe septic shock with multi-organ failure is established, outcomes remain poor.

Unless the infection source is rapidly controlled and the dysregulated inflammatory response abates, even the best supportive technologies cannot prevent progression to death. Our data reinforce this sobering reality. Patients who died had markedly higher illness severity on admission, as reflected by APACHE II scores, consistent with the strong prognostic value of acute severity scores in critical illness [28]. Indeed, the acute physiologic derangements captured by APACHE II and similar indices are well-known to correlate with mortality risk in sepsis. Furthermore, prior studies have confirmed that patients meeting strict Sepsis-3 septic shock criteria suffer very high mortality [29]. In our cohort, the need for CRRT frequently went hand-in-hand with other organ failures in non-survivors, underlining that those who required dialysis were often the sickest patients rather than CRRT itself being a causal factor. When fulminant multi-organ failure sets in, survival becomes unlikely. One manifestation of this “point of no return” is the development of sepsis-induced coagulopathy, which is associated with extremely high mortality [30]. Essentially, the dominant driver of outcome in septic shock is the severity of the underlying illness and ensuing organ dysfunction, more so than any single adjunctive treatment.

Another likely explanation for why layering additional therapies has not translated into major survival gains is the lack of synergistic benefit from intervention stacking. Simply adding more treatments on top of optimized standard care may provide diminishing returns unless those treatments address a specific, uncorrected aspect of the patient’s pathology. Many adjuncts targeting various components of sepsis pathophysiology have failed to improve outcomes in randomized trials. For example, high-dose vitamin C therapy (often combined with thiamine and corticosteroids) did not significantly improve organ failure or mortality in multiple RCTs—including the CITRIS-ALI, VITAMINS, VICTAS, and LOVIT trials [31,32,33,34]. The inability of such interventions to confer a survival advantage suggests that once fundamental resuscitation and supportive care are in place, additional general therapies offer limited benefit. This lack of additive effect indicates that a one-size-fits-all approach with multiple adjuncts is ineffective; instead, a more tailored strategy is needed to achieve meaningful outcome improvements.

Sepsis is a highly heterogeneous syndrome, which further complicates efforts to find universally effective therapies. Shock can arise from different immunopathological pathways—endotoxin-driven vasoplegia is only one subset. If an adjunct like PMX-HP is applied without regard to a patient’s biological phenotype, any true benefit may be diluted [22]. In fact, septic shock comprises distinct clinical endotypes with divergent immune responses and outcomes [35,36]. Recent big-data studies have identified sepsis phenotypes that respond differently to treatments. Outcomes likely depend on delivering the right intervention to the right patient at the right time. For instance, extracorporeal endotoxin removal might benefit a subgroup of patients with overwhelmingly endotoxin-mediated shock (such as fulminant abdominal Gram-negative sepsis) when instituted early, but it may provide little value in others. Similarly, immunomodulatory therapies could help patients with certain host-response profiles but not in all septic patients. In short, the heterogeneity of sepsis means that indiscriminate use of any single therapy will inevitably yield mixed results. Personalized, phenotype-driven approaches are required to realize significant mortality reductions. Developing reliable biomarkers or clinical criteria to identify the subgroups most likely to benefit from specific adjuncts is a key research priority.

Two findings from our study merit particular emphasis. First, the requirement for CRRT was associated with more severe illness and higher crude mortality, but after accounting for illness severity and shock parameters, CRRT itself was not an independent predictor of death. While the crude mortality rate was substantially higher in patients requiring CRRT, this finding should not be interpreted as a harmful effect of CRRT. Rather, the need for CRRT reflects the severity of underlying illness and extent of organ dysfunction. Even with CRRT support, patients with septic AKI had poor outcomes, highlighting the challenge of reversing multi-organ failure once established. This suggests that needing CRRT is primarily a marker of the severity of the patient’s condition (especially the degree of AKI and multi-organ failure) rather than a modifiable risk factor. This suggests that needing CRRT is primarily a marker of the severity of the patient’s condition (especially the degree of AKI and multi-organ failure) rather than a modifiable risk factor. Our observation aligns with evidence from recent CRRT timing trials indicating that earlier or more aggressive initiation of RRT in sepsis does not improve survival and can lead to unnecessary complications [15]. It also resonates with analyses of advanced CRRT membranes: while high-adsorption hemofilters (like oXiris) have shown reductions in vasopressor requirements and short-term mortality in some reports, those studies are heterogeneous and of low certainty [14,24]. Taken together, these data support the view that CRRT should be used to support renal function and fluid balance when indicated, but not with the expectation of directly improving sepsis survival. Clinicians should recognize that patients requiring CRRT are severely ill and focus on treating the underlying septic process, rather than assuming CRRT will alter the disease trajectory.

Second, the trajectory of vasoactive-inotropic score proved to be highly prognostic in our cohort. Patients whose vasopressor requirements failed to decrease after PMX-HP—manifesting as a positive ΔVIS (increasing VIS) over the 24 h post-treatment—had significantly higher mortality. An increasing VIS essentially signified refractory shock despite all ongoing therapies. This finding mirrors prior literature identifying VIS as a robust early indicator of mortality in adult sepsis [16]. It underscores that persistent vasoplegia and inability to wean vasopressors is a warning sign of treatment failure. Even interventions that can transiently raise blood pressure (e.g., angiotensin II infusion) have not shown substantial outcome benefits if the underlying shock state is not resolving [21]. In practice, trending the VIS can serve as a practical bedside tool to gauge a patient’s trajectory. If the VIS is not decreasing within 24–48 h—or worse, is rising—it should prompt clinicians to re-evaluate the adequacy of source control and antimicrobial therapy, consider escalation to additional rescue therapies (such as ECMO or other experimental treatments), and initiate goals-of-care discussions with the patient’s family given the high risk of death. Early identification of a non-improving VIS trajectory may allow timely adjustments in care that could potentially improve outcomes for some patients.

### 4.1. Clinical Implications

Our findings have several implications for the management of septic shock, particularly in settings similar to our East Asian ICU practice:

Targeted use of PMX-HP: The modest and inconsistent impact of PMX-HP on outcomes suggests that this therapy should be used in a targeted fashion rather than routinely. It may be reasonable to consider PMX-HP early in the course of septic shock for patients with a high probability of endotoxin-driven shock—for example, those with Gram-negative bacteremia from abdominal sources who fulfill criteria for high endotoxin activity—Recent reviews by Kellum and Ronco [37] further emphasize that endotoxin plays a central role in a subset of septic shock, and that therapies such as PMX-HP may be most beneficial when applied to patients with demonstrably high endotoxin activity. This supports a precision approach rather than broad application of PMX-HP in all cases.

Optimizing fundamental care: Rigorous adherence to evidence-based sepsis care remains the cornerstone upon which any adjunctive therapies are added. This includes prompt antibiotic administration and source control, lung-protective ventilation for acute respiratory distress syndrome, and other bundle elements. Additionally, supportive care should be optimized: use balanced crystalloid solutions for initial fluid resuscitation instead of normal saline (to reduce the risk of hyperchloremic metabolic acidosis and renal stress) and avoid excessive fluid administration beyond initial stabilization—a conservative fluid strategy after the acute phase has been shown to be safe in septic shock patients. In our practice, once patients are no longer hypotensive, we aim to prevent fluid overload by using diuretics or CRRT for controlled de-resuscitation. Furthermore, meticulous supportive care such as maintaining adequate nutrition, preventing ICU complications, and engaging early rehabilitation can improve overall outcomes and should not be overlooked [38]. Every additional therapy (like PMX-HP) should be seen as a supplement to, not a replacement for, these fundamental measures.

Dynamic monitoring and escalation: We suggest incorporating dynamic metrics like the VIS into sepsis management protocols. Monitoring the trend in vasoactive support requirements can provide an early warning of a patient’s trajectory. For instance, if a patient’s VIS remains high or rises 12–24 h into treatment despite standard care, this should trigger a reassessment and possible escalation. Escalation steps might include deploying adjunctive therapies (such as corticosteroids, if not already used, or investigational treatments available in trial settings), consulting for extracorporeal support (e.g., VA-ECMO in cases of combined cardiac depression and vasoplegia), or considering experimental therapies on compassionate grounds. At the same time, persistent shock should prompt physicians to have frank discussions with families about the prognosis. In selected cases, transitioning goals of care might be appropriate when the likelihood of recovery becomes exceedingly low. Importantly, recent evidence suggests that in older critically ill patients, maintaining a slightly lower blood pressure target (MAP in the low 60s mmHg, as in the 65 trial) is non-inferior to higher targets and can reduce vasopressor exposure [26]. Thus, for patients who are chronically hypertensive and elderly, tolerating a lower blood pressure (with careful monitoring of organ perfusion) might be acceptable and could shorten the duration of vasopressor support needed.

Role of CRRT: Our results reinforce that CRRT should be viewed primarily as a supportive measure for renal failure and fluid management, rather than a therapy that will improve sepsis survival. Clinicians should initiate CRRT for the standard indications (e.g., significant azotemia, oliguria with volume overload, severe metabolic derangements) and not purely in an attempt to “filter out” inflammatory mediators. Early initiation of CRRT in the absence of life-threatening renal indications is not supported by evidence [15]. In practice, we start CRRT in septic shock patients when there is persistent oliguria with volume overload or hyperkalemia/acidosis that does not respond to medical management. We do not start CRRT solely due to high cytokine levels or in hopes of modulating sepsis, because studies have not shown a mortality benefit to doing so. That said, when CRRT is used, certain advanced membranes (e.g., high-adsorption filters) might confer hemodynamic stabilization by adsorbing some mediators [14], but such benefits are adjunctive and not a substitute for treating the infection. Ultimately, the presence of CRRT requirement signals a very sick patient—which should prompt vigilance and aggressive primary sepsis therapy—but instituting CRRT earlier or more intensively is not proven to independently improve survival.

### 4.2. Strengths and Limitations

Strengths of this study include the detailed hemodynamic profiling (using VIS trajectories) and integration of organ support timing in analyzing outcomes, as well as the focus on a regional patient population under real-world practice and reimbursement conditions—aspects that are often under-represented in large randomized trials. We captured granular data on vasopressor changes around PMX-HP therapy, which allowed us to identify VIS dynamics as a key prognostic indicator. Additionally, our cohort reflects a practical clinical setting in East Asia where PMX-HP is available and reimbursed, thereby providing insight into real-world utilization and outcomes outside of strictly controlled trial environments.

However, several limitations merit consideration. First, the retrospective single-center design carries risks of residual confounding and selection bias (e.g., the sickest patients were more likely to receive PMX-HP and CRRT by clinical decision). Our sample size was modest (n = 64), limiting power to detect small-to-moderate effect sizes in multivariable analyses. We did not measure endotoxin activity levels, so we could not directly correlate endotoxemia burden with response to PMX-HP—it is possible that some patients did not have high endotoxin levels and thus were less likely to benefit. Long-term outcomes beyond hospital discharge were not captured, so the impact on longer-term survival or quality of life remains unknown; this is an important gap given the challenges many sepsis survivors face in recovery [17]. We also focused on patients who met Sepsis-3 septic shock criteria and received PMX-HP, which may limit generalizability to less severe sepsis cases—our findings may not apply to patients with sepsis who never develop refractory shock or to settings where PMX-HP is not used. In addition, temporal changes in sepsis care over the study period (e.g., adoption of more conservative fluid strategies or vasopressor-sparing approaches after 2018) could influence the generalizability across different time frames. Finally, because this was a retrospective, observational study, we can only report associations and cannot conclusively establish causation for the effects of CRRT or PMX-HP on outcomes.

### 4.3. Future Directions

Improving outcomes in septic shock will likely require personalized and timely approaches—a move toward “precision sepsis” care. Key avenues for future research and quality improvement include: (i) Biomarker-guided and phenotype-driven therapies: Developing rapid diagnostics (such as endotoxin activity assays or host-response biomarker panels) to identify which patients might benefit from specific adjuncts like PMX-HP and deploying those therapies within hours of shock onset for maximum effect. Emerging data-driven models have identified novel sepsis phenotypes that could be targeted in tailored trials [39]. (ii) Protocolized escalation tied to early trajectory markers: Combining standard sepsis bundles with decision triggers (e.g., failure of VIS or lactate to improve after a set time) to prompt escalation or alteration of therapy. For example, a protocol might stipulate that if VIS remains >20 after 24 h, an interdisciplinary review is required to consider additional interventions or to evaluate for unrecognized problems in source control. (iii) Immunomodulation and adjunctive therapies: Investigating treatments that address the immune dysfunction of sepsis—for instance, therapies targeting immune checkpoints or aiming to restore immune homeostasis in the later phases of sepsis [40]. Early-phase trials of immunoadjuvant therapies (such as anti-PD1/PDL1 agents or IL-7 for septic patients with immune exhaustion) are underway. Additionally, strategies to improve long-term outcomes for sepsis survivors, such as structured rehabilitation and follow-up programs, warrant attention [41]. By refining patient phenotyping, improving real-time monitoring of illness trajectory, and exploring adjuncts that either modulate the immune response or support failing organs more effectively, future research may unlock meaningful reductions in sepsis mortality and morbidity.

## 5. Conclusions

Sepsis remains a global health priority, and even mandated early sepsis care protocols have yielded only modest improvements in outcomes. In this retrospective cohort of critically ill patients with septic shock receiving PMX-HP therapy, the overall 28-day mortality remained high (~47%). The need for CRRT—largely reflecting severe AKI and greater illness severity—was associated with worse unadjusted outcomes but was not an independent predictor of mortality in adjusted analysis. In contrast, patients whose hemodynamics failed to improve (VIS increased) after PMX-HP had significantly higher mortality. These findings suggest that outcomes in refractory septic shock are driven principally by underlying disease severity and persistent organ failures (especially refractory vasodilatory shock and multi-organ failure), rather than by the addition of any single adjunctive therapy. Careful patient selection for therapies like PMX-HP, early identification of patients with rising VIS who are not responding to conventional treatment, and excellence in supportive care (including timely initiation of CRRT when indicated and strict adherence to core sepsis bundles) are essential. Further multicenter studies are needed to determine optimal patient selection and timing for interventions such as PMX-HP and to clarify the interplay of CRRT, immunomodulation, and extracorporeal blood purification in modern sepsis management.

## Figures and Tables

**Table 1:** Baseline characteristics of the patients receiving PMX-HP (n = 64).

Characteristic	All Patients (n = 64)
Age, years	66.1 ± 12.3
Male sex, n (%)	43 (67.2%)
Body weight, kg	66.5 ± 15.2
APACHE II score	26 (21–32)
Comorbidities: COPD/asthma, n (%)	7 (10.9%)
Congestive heart failure, n (%)	15 (23.4%)
Liver cirrhosis, n (%)	9 (14.1%)
History of stroke, n (%)	8 (12.5%)
Diabetes mellitus, n (%)	32 (50.0%)
Hypertension, n (%)	35 (54.7%)
Active cancer, n (%)	12 (18.8%)
Chronic kidney disease/ESRD, n (%)	26 (40.6%)
Source: Pneumonia, n (%)	19 (29.7%)
Source: Intra-abdominal, n (%)	16 (25.0%)
Source: Urinary tract, n (%)	11 (17.2%)
Source: Others, n (%)	18 (28.1%)

Abbreviations: PMX-HP, polymyxin-B hemoperfusion; APACHE, Acute Physiology and Chronic Health Evaluation; COPD, chronic obstructive pulmonary disease; ESRD, end-stage renal disease.

**Table 2:** Clinical outcomes of patients treated with PMX-HP.

Outcome	All Patients (n = 64)
28-day mortality, n (%)	30 (46.9%)
ICU mortality, n (%)	30 (46.9%)
Hospital mortality, n (%)	34 (53.1%)
ICU length of stay, days	9.3 (4.4–21.1)
Hospital length of stay, days	20.5 (8.0–34.3)
CRRT use, n (%)	43 (67.2%)
ECMO use, n (%)	3 (4.7%)

Abbreviations: PMX-HP, polymyxin-B hemoperfusion; ICU, intensive care unit; CRRT, continuous renal replacement therapy; ECMO, extracorporeal membrane oxygenation.

**Table 3:** Comparison of survivors vs. non-survivors at 28 days.

Variable	Survivors (n = 34)	Non-Survivors (n = 30)	*p*-Value
Age, years	64.3 ± 13.8	68.2 ± 10.3	0.200
Male sex, n (%)	20 (58.8%)	23 (76.7%)	0.210
APACHE II score	25 (20–28)	28 (23–33)	0.100
Source—UTI, n (%)	10 (29.4%)	1 (3.3%)	0.020
Source—Others, n (%)	2 (5.9%)	8 (26.7%)	0.040
CRRT use by 28 days, n (%)	19 (55.9%)	24 (80.0%)	0.048
ECMO use, n (%)	1 (2.9%)	2 (6.7%)	0.600
PMX sessions (median, IQR)	2 (2–2)	2 (1–2)	0.080
Increase in VIS after PMX, n (%)	2 (5.9%)	12 (40.0%)	<0.010

Abbreviations: APACHE, Acute Physiology and Chronic Health Evaluation; CRRT, continuous renal replacement therapy; ECMO, extracorporeal membrane oxygenation; UTI, urinary tract infection; VIS, vasoactive-inotropic score.

**Table 4:** Univariate and multivariate analysis of risk factors for 28-day mortality.

Variable	Univariate OR (95% CI)	Multivariate OR (95% CI)	*p*-Value
APACHE II (per point)	1.09 (1.00–1.18)	1.08 (0.99–1.18)	0.070
CRRT use (yes vs. no)	3.2 (0.9–11.0)	2.5 (0.7–9.0)	0.150
ΔVIS increase (yes vs. no)	10.7 (2.2–51.5)	8.4 (1.8–39.5)	0.003

Abbreviations: APACHE, Acute Physiology and Chronic Health Evaluation; CRRT, continuous renal replacement therapy; VIS, vasoactive-inotropic score.

## Data Availability

The patient data are stored in the hospital clinical database and are not publicly available due to privacy regulations. Anonymized data may be obtained from the corresponding author upon reasonable request.
